# The Role of the Parkinson's Disease Gene PARK9 in Essential Cellular Pathways and the Manganese Homeostasis Network in Yeast

**DOI:** 10.1371/journal.pone.0034178

**Published:** 2012-03-23

**Authors:** Alessandra Chesi, Austin Kilaru, Xiaodong Fang, Antony A. Cooper, Aaron D. Gitler

**Affiliations:** 1 Department of Genetics, Stanford University School of Medicine, Stanford University, Stanford, California, United States of America; 2 Department of Cell and Developmental Biology, Perelman School of Medicine, University of Pennsylvania, Philadelphia, Pennsylvania, United States of America; 3 Garvan Institute of Medical Research, University of New South Wales, Sydney, New South Wales, Australia; Hertie Institute for Clinical Brain Research and German Center for Neurodegenerative Diseases, Germany

## Abstract

YPK9 (Yeast PARK9; also known as YOR291W) is a non-essential yeast gene predicted by sequence to encode a transmembrane P-type transport ATPase. However, its substrate specificity is unknown. Mutations in the human homolog of YPK9, ATP13A2/PARK9, have been linked to genetic forms of early onset parkinsonism. We previously described a strong genetic interaction between Ypk9 and another Parkinson's disease (PD) protein α-synuclein in multiple model systems, and a role for Ypk9 in manganese detoxification in yeast. In humans, environmental exposure to toxic levels of manganese causes a syndrome similar to PD and is thus an environmental risk factor for the disease. How manganese contributes to neurodegeneration is poorly understood. Here we describe multiple genome-wide screens in yeast aimed at defining the cellular function of Ypk9 and the mechanisms by which it protects cells from manganese toxicity. In physiological conditions, we found that Ypk9 genetically interacts with essential genes involved in cellular trafficking and the cell cycle. Deletion of Ypk9 sensitizes yeast cells to exposure to excess manganese. Using a library of non-essential gene deletions, we screened for additional genes involved in tolerance to excess manganese exposure, discovering several novel pathways involved in manganese homeostasis. We defined the dependence of the deletion strain phenotypes in the presence of manganese on Ypk9, and found that Ypk9 deletion modifies the manganese tolerance of only a subset of strains. These results confirm a role for Ypk9 in manganese homeostasis and illuminates cellular pathways and biological processes in which Ypk9 likely functions.

## Introduction

Ypk9 (YOR291W) is a non-essential gene in the budding yeast, *Saccahromyces cerevisiae*, predicted by sequence to belong to a family of cationic P-type transport ATPases of unknown substrate specificity. Recently, mutations in the human homolog of Ypk9, ATP13A2/PARK9, have been linked to Parkinson's disease (PD) and PD-like Kufor-Rakeb syndrome [1]. PD is associated with a spectrum of diverse genetic and environmental susceptibilities [2]. α-Synuclein (α-syn) is the best studied of the genetic factors, with point mutations in its coding sequence or duplications and triplications of its entire locus causing early onset autosomal dominant forms of familial PD [3], [4], [5]. α-Syn plays a key role in sporadic PD as well, being the major component of Lewy bodies, the proteinaceous inclusions characteristic of the disease [6]. Expression of α-syn in multiple model systems including yeast, worm, fly, and mouse is sufficient to cause neurodegeneration, and simple experimental model systems have proven to be an invaluable tool to gain insight into basic cellular mechanisms by which α-syn might contribute to disease [7], [8], [9], [10].

When expressed in yeast at low levels, α-syn localizes to the plasma membrane, consistent with its presynaptic localization in neurons. Increasing the level of expression causes a growth defect and relocalization of α-syn to cytoplasmic inclusions composed of clusters of mislocalized transport vesicles from various steps of the endocytic and exocytic pathways [11], [12], [13].

We previously identified Ypk9 as a potent modifier of α-syn toxicity in an unbiased yeast genetic screen [14], [15]. Importantly, the ability of Ypk9 to rescue α-syn toxicity is conserved and upregulation of the mammalian PARK9 gene is sufficient to rescue α-syn-induced neurodegeneration in multiple animal models [14]. However, the mechanism by which Ypk9 protects against α-syn toxicity is unknown. Therefore, defining the normal cellular function of Ypk9 will provide insight into how it is able to mitigate α-syn toxicity as well as how its loss of function might contribute to PD.

Subcellular localization studies have revealed that Ypk9 is localized to the vacuolar membrane in yeast [14], analogous to the lysosomal localization of ATP13A2 in mammalian cells [1]. Proper vacuolar localization and ATPase activities are both required for Ypk9 to protect against α-syn toxicity, since incorrectly localized patient-based truncation mutants or an ATPase-dead mutant (D781N) are unable to rescue α-syn toxicity [14].

Although Ypk9 is not an essential gene in yeast under normal conditions, *ypk9*Δ cells are selectively hypersensitive to an excess of manganese ions in the culture media [14], [16]. Accordingly, overexpression of Ypk9 protects cells from manganese toxicity, while PD-linked mutations or the ATPase-dead mutant D781N do not protect [14]. Recently, similar findings have been reported for the human ortholog, ATP13A2/PARK9, in mammalian cell lines [17], confirming the value of the yeast model system as a tool to study genes and pathways relevant to human disease [18].

Manganese (Mn^2+^) is a trace element required as an essential nutrient by all known organisms. Mn^2+^ ions are a key cofactor for a large variety of enzymes present in virtually every compartment of the cell, such as oxidases and dehydrogenases, DNA and RNA polymerases, kinases, decarboxylases, sugar transferases, and superoxide dismutases [19]. At high concentrations, however, manganese is toxic. In humans, for instance, exposure to manganese has been associated with a neurological syndrome called manganism, whose symptoms resemble those of PD [20], [21]. For these reasons, control of manganese trafficking and handling is critical for the cell and must be carefully regulated by cellular manganese homeostasis pathways, such as cell surface and intracellular manganese transporters, manganese chaperones and detoxification factors that sequester or eliminate Mn^2+^ from the cell [19].

Much of our current understanding of manganese homeostatic mechanisms comes from genetic studies of the budding yeast [22]. Outside of the physiological range of intracellular manganese, yeast cells are stressed by Mn^2+^ deficiency or toxicity, and respond by upregulating or downregulating cell surface and intracellular transport systems to normalize intracellular manganese levels [22]. Regulation of manganese transport in yeast does not involve any known transcriptional pathway, such as those described for transporters of copper, zinc, and iron. Instead, it occurs at the post-translational level through changes in transporter protein localization and turnover [23].

The Nramp Smf1 and Smf2 transporters are largely responsible for uptake of essential manganese (Smf2) and for oxidative stress protection (Smf1). Under Mn^2+^ replete conditions, ∼90% of Smf1 and Smf2 are targeted to the vacuole for degradation, presumably to limit uptake of toxic amounts of metals. When cells are starved for manganese, the polypeptides are not delivered to the vacuole and localize instead to the cell surface (Smf1) and intracellular vesicles (Smf2) to increase Mn^2+^ uptake. In conditions of toxic metal excess, vacuolar degradation of the Nramp transporters is enhanced, virtually eliminating Smf1p from the cell [23].

Under manganese surplus conditions, the metal can enter the cell by an additional route, specifically through the high-affinity cell surface phosphate transporter Pho84. Accordingly, mutations in Pho84 and in another yeast gene involved in phosphate metabolism, Vtc4, are associated with manganese resistance and lowering of intracellular manganese [24].

An important route of manganese detoxification in yeast is through Pmr1, a Golgi-localized P-type Ca^2+^ and Mn^2+^ ATPase, which sustains the processing and trafficking of proteins moving through the secretory pathway. Pmr1 provides manganese, an essential cofactor for protein glycosylation, to sugar transferases, and transports toxic surplus manganese into the secretory pathway for excretion [25].

Another route of manganese detoxification in yeast is the vacuole, a major site for toxic metal ion storage. Thus far, only one vacuolar manganese (and iron) transporter has been definitively identified, Ccc1 [26]. Interestingly, Ccc1 was also identified as a multi-copy suppressor of α-synuclein toxicity in the yeast genetic screen [15].

Although a few genes involved in Mn^2+^ homeostasis have been identified in yeast, our knowledge of the Mn^2+^ homeostasis network is still limited. Since Ypk9 is localized to the vacuole, the site of sequestration of excess Mn^2+^ in yeast cells, and *ypk9*Δ cells are hypersensitive to Mn^2+^, Ypk9 is a good candidate manganese homeostasis gene. In order to define the cellular function of Ypk9 and the mechanism by which it protects cells from Mn^2+^ toxicity, we took advantage of the power of the yeast system and performed multiple genetic screens to identify genes and cellular pathways that interact with Ypk9, in standard culture conditions or in presence of subtoxic concentrations of Mn^2+^. These studies provide a comprehensive list of yeast genes involved in manganese homeostasis. Interestingly, we found that some of these manganese homeostasis genes require Ypk9 function whereas others do not, indicating a role for Ypk9-dependent and -independent pathways in protecting against manganese toxicity. These findings pave the way to functional studies in mammalian systems, providing a framework for deciphering how genetic and environmental factors might interact to contribute to PD and other related disorders.

## Results

### Ypk9 interacts with essential yeast genes

The Ykp9 deletion strain is viable and shows no growth defect under standard culture conditions ([14] and A.D.G. and A.C. unpublished observations). To provide insight into Ypk9 cellular function, we took advantage of the yeast system and performed a genome-wide synthetic lethal screen. The yeast genome contains ∼6,000 genes and ∼4,850 of these are non-essential. We used the synthetic genetic array (SGA) technique to introduce by mating a Ypk9 deletion (*ypk9Δ*) into each yeast deletion strain of two different libraries: a deletion library of non-essential yeast genes [27] and a library of ∼1,335 temperature sensitive (TS) mutants of essential genes [28], [29]. Following sporulation, we selectively germinated meiotic progeny containing both the Ypk9 and the gene deletions. We compared growth of each strain on agar plates containing G418 (single deletion) to that on G418 plus nourseothricin (double deletion). The phenotype we used in all of these screens was colony size after growth for three days. Both the non-essential gene deletions and the TS mutant strains (at the permissive temperature, 25°C) showed no significant growth defect at baseline conditions.

Following multiple rounds of screening, we did not identify any synthetic sick or lethal (SSL) interaction with any gene in the non-essential deletion collection. Instead, we identified 12 synthetic sick interactions with essential genes (Table 1). The 12 double KOs hits showed more than 50% reduction in colony size compared to the average colony size of the plate. We repeated the screen three independent times and we selected only the hits that reproduced all three times. Furthermore, many of the hits were identified more than once (because the same strain was duplicated on different plates in the library) or different mutant alleles of the same gene were identified.

**Table 1:** Essential yeast genes that show synthetic sick interactions with Ypk9.

ts allele	Yeast Gene	Human Homolog	Function
alg1-1	ALG1	ALG1	Mannosyltransferase
cdc28-td	CDC28	CDK2	Catalytic subunit of the main cell cycle cyclin-dependent kinase (CDK)
cdc2-1	POL3	POLD1	Catalytic subunit of DNA polymerase delta
cdc53-1	CDC53	CUL1	Cullin, structural protein of SCF complexes
cdc12-td	CDC12	SEPT4	Component of the septin ring of the mother-bud neck that is required for cytokinesis
cdc11-4, cdc11-5	CDC11	no	Component of the septin ring of the mother-bud neck that is required for cytokinesis
sfh1-1	SFH1	SMARCB1	Component of the RSC chromatin remodeling complex
gab1-1	GAB1	PIGU	GPI transamidase subunit
apc5-CA-PAps	APC5	no	Subunit of the Anaphase-Promoting Complex/Cyclosome (APC/C), which is a ubiquitin-protein ligase
myo2-16	MYO2	MYO5C	One of two type V myosin motors
dim1-2	DIM1	DIMT1L	Essential 18S rRNA dimethylase, involved in pre-ribosomal RNA processing
bet2-1	BET2	RABGGTB	Beta subunit of Type II geranylgeranyltransferase required for vesicular transport between the endoplasmic reticulum and the Golgi

Most of the hits have a clear human homolog, and are involved in the cell cycle (APC5, CDC28, CDC53, SFH1, POL3, CDC11, CDC12), cellular transport and vesicular trafficking (ALG1, GAB1, BET2, MYO2), or RNA processing (DIM1). One interesting deletion is BET2, which encodes the beta subunit of the type II geranylgeranyl transferase required for vesicular transport between the endoplasmic reticulum and the Golgi, and provides a membrane attachment moiety to the Rab-like proteins Ypt1 and Sec4 [30], [31]. Interestingly, overexpression of either Ypt1 or Ypk9 is sufficient to rescue yeast cells from α-synuclein toxicity, by relieving the block in the ER-to-Golgi trafficking caused by α-synuclein overexpression [13], and the two genes show a synergistic interaction when co-expressed [14]. Taken together, these data suggest potential roles for Ypk9 in cell cycle and vesicular trafficking.

Other notable hits from this screen are the septins CDC11 and CDC12. Septins are required for cytokinesis in many species [32]. Mammalian septins are abundantly expressed in the CNS, where they have been implicated in exocytosis [33], [34]. Septins have also been implicated in diverse neurodegenerative disorders in humans and, intriguingly, in PD and α-synuclein mediated toxicity. Sept4 has been found co-localized with α-synuclein in cytoplasmic inclusions known as Lewy bodies (LBs) in sporadic PD and dementia with Lewy bodies and in glial cytoplasmic inclusions in multiple system atrophy [35]. Sept4 and Sept5/CDCrel-1 are substrates for a ubiquitin ligase, parkin, which is another familial PD gene; loss of parkin function may result in inappropriate accumulation of these paralogous septins [36], [37], [38]. An acute overload of Sept5 can be both inhibitory to exocytosis and neurotoxic [33], [38], [39]. While mice that lack Sept3, Sept5 or Sept6 do not show obvious CNS abnormalities [40], [41], [42], Sept4 KO mice show a specific attenuation of nigrostriatal DA transmission [43]. Moreover, genetic loss of Sept4 exacerbates neuronal loss, gliosis and locomotor deterioration in transgenic mice expressing human “aaß”a-synuclein A53T [43]. It is tempting to speculate that septins and Ypk9 may participate in the same cellular pathway, and that loss of both septin and Ypk9 function might synergize, eventually exacerbating α-synuclein toxicity.

### A genome-wide screen identifies yeast genes involved in manganese tolerance

**Figure 1:**
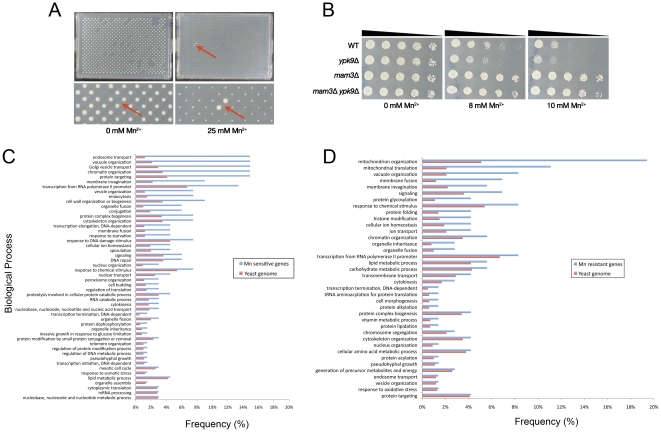
Yeast genetic screens provide insight into the manganese homeostasis network. **A**, Screen for manganese resistant deletion strains. A representative plate is shown (each plate contains 384 different yeast deletion strains pinned in duplicate, 768 total colonies). Left, control plate (0 mM Mn^2+^); right, plate containing 25 mM Mn^2+^. The red arrow points to a manganese resistant strain, growing better than the other strains in the presence of a toxic concentration of Mn^2+^. **B**, Manganese resistance in the *mam3Δ* strain is independent of Ypk9 function. A representative spotting assay showing that resistance to Mn^2+^ in the *mam3*Δ strain is independent of Ypk9. Five-fold serial dilutions of WT, *ypk9*Δ, *mam3*Δ and *mam3*Δ *ypk9*Δ were spotted on SD plates containing different Mn^2+^ concentrations. **C**, Functional categories of genes enriched as manganese sensitive hits in the Mn^2+^ tolerance screen compared to the yeast genome. **D**, Functional categories of genes enriched as manganese resistant hits in the Mn^2+^ tolerance screen compared to the yeast genome.

We previously identified Ypk9 as a gene that has a role in protecting yeast cells from manganese (Mn^2+^) toxicity [14]. Although some genes involved in Mn^2+^ tolerance have been reported [44], [45], [46], [47], to our knowledge, there are no published genome-wide studies defining a comprehensive yeast Mn^2+^ homeostasis network. We therefore performed a screen, spotting a collection of ∼4,850 non-essential yeast gene deletion strains onto plates containing two different concentrations of Mn^2+^ (Fig. 1A). We used a lower concentration (12 mM) to identify deletion strains that were hypersensitive to Mn^2+^ toxicity, and a higher concentration (25 mM) to identify resistant strains. The baseline phenotypes on 12 mM and 25 mM Mn^2+^ plates were a reduction in colony size of 34% and 76%, respectively. Deletion of the non-essential genes indicated as “sensitive” hits caused a growth defect much bigger (colony size less than 50% of the average colony size in the plate) than the baseline, while deletion of the “resistant” hits caused a smaller or no growth defect (colony size more than 175% of the average colony size in the plate). We identified 67 deletion strains that were sensitive and 72 that were resistant to Mn^2+^ toxicity in three independent rounds of screening, many with clear human homologs (Tables S1 and S2 in Online Supporting Information).

The Mn^2+^ sensitive hits were enriched in the main functional categories of vesicle-mediated transport, vacuolar organization and chromatin remodeling (see Fig. 1C for a complete list of enriched gene ontology (GO) terms). The first two categories are expected since, in *S. cerevisiae*, the main routes of detoxification for Mn^2+^ (as for other toxic metals) are the secretory pathway (through the Golgi) and the vacuole [22]. All the different steps in vesicular transport (endosome, vacuole, Golgi) seem to be important for Mn^2+^ detoxification. In particular, we identified all the components of the GARP (Golgi-associated retrograde protein) complex (VPS51, VPS52, VPS53, VPS54) as Mn^2+^ sensitive deletions, together with the Rab GTPase YPT6 and its GTP exchange factor RGP1/RIC1. These genes work together in the retrograde transport from endosome to Golgi, which is required for the recycling of membrane proteins and for vacuolar protein sorting [48]. Interestingly, mutations in VPS35, a component of the retromer complex, involved in retrograde transport, were recently reported as a cause of late-onset PD [49], [50].

The other highly enriched category is chromatin organization, in particular histone exchange. We identified 8 genes belonging to the SWR1 complex (SWC3, SWC5, SWR1, VPS72, ARP6, VPS71, YAF9, HTZ1) responsible for exchanging histone variant H2AZ (HTZ1) for histone H2A in nucleosomes and involved in transcriptional regulation through prevention of the spread of silent heterochromatin [51], [52].

The Mn^2+^ resistant gene network is enriched in the GO categories of mitochondrial organization/translation, vacuolar organization and membrane fusion (Fig. 1D). Among the most resistant deletion strains, we identified the vacuolar transporter chaperone (VTC) complex members VTC1, VTC2 and VTC3, involved in membrane trafficking, vacuolar polyphosphate accumulation, microautophagy and non-autophagic vacuolar fusion [53], [54], [55], [56], and the class II histone deacetylase complex members HDA1, HDA2 and HDA3.

These findings expand our knowledge of manganese homeostasis in yeast, confirming the importance of the secretory pathway, the vacuole and the mitochondria in Mn^2+^ homeostasis and detoxification, and suggest a new role for chromatin remodeling and histone exchange/modification pathways.

### Ypk9 deletion modifies manganese tolerance of a subset of genes

Having established a comprehensive set of yeast deletion strains that are either sensitive or resistant to manganese, we next asked whether these phenotypes were dependent on Ypk9 function. To assess this, we performed a genetic epistasis analysis by generating double knockouts (yeast deletion conferring manganese resistance or sensitivity + *ypk9*Δ) and compared Mn^2+^ tolerance of the double mutant strains to baseline phenotypes of each single deletion strain. The baseline phenotypes for the double KOs were normal growth on control plates, and 35% and 83% growth defect on 12 mM and 25 mM Mn containing plates, respectively. The same thresholds described for the single KOs were applied to identify sensitive and resistant double KOs.

The results are summarized in Table 2. Among the 72 resistant deletions, we identified 18 strains that lost resistance to Mn^2+^ upon Ypk9 deletion (aggravating interaction), while 32 did not (the remaining 22 double deletions did not pass quality control; see Methods). Interestingly, we also identified 5 strains that acquired Mn^2+^ resistance upon Ypk9 deletion (alleviating interaction). Among the 67 sensitive deletions, we identified 3 strains that lost sensitivity to Mn^2+^ (alleviating interaction) and 16 that did not (48 did not pass quality control). We identified 10 strains that acquired sensitivity to Mn^2+^ upon Ypk9 deletion (aggravating interaction). We validated the screen results of selected hits by secondary spotting assays on plates containing different Mn^2+^ concentrations (Fig. 1B).

**Table 2:** Deletion strains whose Mn^2+^ tolerance is dependent or independent on Ypk9.

*Mn^2+^ Resistant genes*				*Mn^2+^ Sensitive genes*			
*Ypk9-independent*		*Ypk9-dependent*		*Ypk9-independent*		*Ypk9-dependent*	
Systematic Name	Common Name	Systematic Name	Common Name	Systematic Name	Common Name	Systematic Name	Common Name
YLR056W	ERG3	*YDR298C*	*ATP5*	YPL259C	APM1	*YPR051W*	*MAK3*
YLL029W	FRA1	*YFL007W*	*BLM10*	YBR164C	ARL1	*YER122C*	*GLO3*
YHR108W	GGA2	*YHR193C*	*EGD2*	YMR198W	CIK1	*YJR043C*	*POL32*
YNL021W	HDA1	*YDR414C*	*ERD1*	YKL190W	CNB1	*YHR012W*	*VPS29*
YOL060C	MAM3	*YLL049W*	*LDB18*	YNL027W	CRZ1	*YPL196W*	*OXR1*
YPL050C	MNN9	*YDL167C*	*NRP1*	YKR019C	IRS4	*YDL077C*	*VAM6*
YHR004C	NEM1	*YDR289C*	*RTT103*	YKL064W	MNR2	*YOR106W*	*VAM3*
YLR093C	NYV1	*YPL106C*	*SSE1*	YMR123W	PKR1	*YKL113C*	*RAD27*
YCR037C	PHO87	*YPR040W*	*TIP41*	YPL179W	PPQ1	*YDR126W*	*SWF1*
YDR435C	PPM1	*YHR180W*	*YHR180W*	YJR033C	RAV1	*YJL053W*	*PEP8*
YKR028W	SAP190	*YIL054W*	*YIL054W*	YBR231C	SWC5	**YIL040W**	**APQ12**
YER072W	VTC1	*YLL044W*	*YLL044W*	YJL004C	SYS1	**YJR074W**	**MOG1**
YJL012C	VTC4	*YOR199W*	*YOR199W*	YKL081W	TEF4	**YMR031W-A**	**YMR031W-A**
YHR135C	YCK1	*YIL145C*	*PAN6*	YOL018C	TLG2		
YDR089W	YDR089W	*YDL095W*	*PMT1*	YKR001C	VPS1		
YDR295C	HDA2	*YLL030C*	*RRT7*	YJL029C	VPS53		
YPR179C	HDA3	*YIL047C*	*SYG1*				
YPL019C	VTC3	*YEL057C*	*YEL057C*				
YLR087C	CSF1	**YNL148C**	**ALF1**				
YBR014C	GRX7	**YDR174W**	**HMO1**				
YDR297W	SUR2	**YOR123C**	**LEO1**				
YLR372W	SUR4	**YHL023C**	**NPR3**				
YDL122W	UBP1	**YBR103W**	**SIF2**				
YML097C	VPS9						
YML020W	YML020W						
YLR138W	NHA1						
YIL153W	RRD1						
YMR226C	YMR226C						
YMR015C	ERG5						
YDL230W	PTP1						
YGR284C	ERV29						
YGR285C	ZUO1						

In normal-face, Ypk9 independent strains; in italics, strains that lose resistance or gain sensitivity (aggravating interactions); in bold-face, strains that either gain resistance or lose sensitivity (alleviating interactions).

The genes whose Mn^2+^ resistance or sensitive phenotype is independent of Ypk9 likely work in Mn^2+^ homeostasis via different, independent pathways than those of Ypk9. Another possibility is that they may be upstream of Ypk9 in the same pathway (epistatic to Ypk9). An example of this would be a low affinity Mn^2+^ transporter on the plasma membrane, whose deletion confers resistance to Mn^2+^ because the metal cannot enter the cell; Ypk9 deletion would not have any additional effect because Ypk9 is active inside the cell, downstream in the pathway. An example of this kind of interaction that we identified in the screen is PHO87, a membrane phosphate transporter that could function as a low affinity manganese transporter similarly to PHO84, another plasma membrane phosphate transporter that has been shown to play a role in Mn homeostasis [24].

The deletion strains that change their Mn^2+^ tolerance phenotype upon Ypk9 deletion require Ypk9 function to work in Mn^2+^ homeostasis, and these genes are likely to function in the same pathway as Ypk9. Among these, the deletions that become more sensitive or less resistant to Mn^2+^ upon Ypk9 deletion show an aggravating interaction. These hits are enriched in the GO categories of vacuolar and vesicle organization and membrane fusion, supporting a role for Ypk9 in these cellular processes. VAM3, for example, is a vacuolar t-SNARE that mediates docking and fusion of late transport vesicles with the vacuole [57], while VAM6 is required in the tethering steps of vacuolar membrane fusion, and they are both required in the delivery of vacuolar hydrolases [58]. SWF1 is a palmitoyltransferase that acts on SNAREs and may have a role in vacuolar fusion. GLO3 is an ARF GAP involved in ER-Golgi transport [59] that, interestingly, genetically interacts with BET1, which, like BET2 identified by our TS screen, is important for Ypt1 function in vesicular trafficking [30]. The strains that became less sensitive or more resistant upon Ypk9 deletion show an alleviating interaction. These hits are enriched in the GO categories of chromatin organization/histone modification and nuclear transport. In the first category we identified SIF2, a subunit of the Set3C histone deacetylase complex, HMO1, a chromatin associated high mobility group family member involved in genome maintenance, and LEO1, which associates with RNA polymerase II and is involved in histone methylation. APQ12 and MOG1 are instead nuclear proteins involved in RNA and protein transport, respectively.

## Discussion

Mutations in the gene ATP13A3/PARK9 have been recently associated with familial forms of parkinsonism. Interestingly, the yeast homologue gene, Ypk9, protects yeast cells from the toxicity caused by two other insults related to Parkinson's disease, one genetic (α-synuclein overexpression) and one environmental (exposure to high concentrations of manganese). However, Ypk9 is not an essential gene and its normal function is still unclear. To define the cellular function of Ypk9 and the mechanisms by which it protects cells from manganese toxicity, we performed multiple genome-wide screens in yeast.

First, we performed two synthetic lethal screens with a *ypk9*Δ strain, one with a collection of all the ∼4,850 non-essential yeast genes and another with a library of ∼1,335 temperature sensitive mutants of essential genes. We discovered 12 essential genes interacting with Ypk9, involved in different cellular pathways: the cell cycle (APC5, CDC28, CDC53, SFH1, POL3, CDC11, CDC12), cellular transport and vesicular trafficking (ALG1, GAB1, BET2, MYO2), or RNA processing (DIM1). Interestingly, among the hits we identified two members (CDC11 and CDC12) of the septin family. Septins have been implicated in diverse neurodegenerative disorders in humans and, intriguingly, in PD and α-synuclein mediated toxicity [35], [43].

Second, we investigated the role of Ypk9 in manganese homeostasis. To do this, we first identified yeast genes involved in Mn tolerance, spotting a collection of ∼4,850 non-essential yeast gene deletion strains onto plates containing two different concentrations of Mn^2+^, obtaining a comprehensive set of Mn sensitive and resistant deletions. Among the sensitive hits, we found genes involved in vesicle-mediated transport, vacuolar organization and chromatin remodeling, in particular all the components of the GARP complex and the Rab GTPase YPT6 and its GTP exchange factor RGP1/RIC1, and 8 genes belonging to the SWR1 complex responsible for exchanging histone variant H2AZ (HTZ1) for histone H2A in nucleosomes. The resistant hits were enriched in the GO categories of mitochondrial organization/translation, vacuolar organization and membrane fusion, with the vacuolar transporter chaperone (VTC) complex members VTC1, VTC2 and VTC3 among the most resistant deletion strains. To our knowledge, this is the first complete genome-wide investigation of Mn homeostasis genes in yeast. These results will provide important clues for a better understanding of Mn biology in yeast.

With this comprehensive set of Mn homeostasis genes in hand, we then asked which of these might function with Ypk9 and which of these function in independent pathways. We generated *ypk9Δ geneX*Δ double deletions and assessed the effect on Mn tolerance. This allowed us to classify yeast Mn homeostasis genes as Ypk9-dependent or independent. Among the Ypk9-dependent hits, the aggravating interactors (such as VAM3, VAM6, SWF1 and GLO3) were enriched in the categories of vacuolar and vesicle organization and membrane fusion, supporting a role for Ypk9 in these cellular processes. The alleviating interactors (such as SIF2, HMO1, LEO1, APQ12 and MOG1) were enriched in the categories of chromatin organization/histone modification and nuclear transport.

Taken together, these results confirm a role for Ypk9 in manganese tolerance, identify candidate genes and cellular processes that interact with Ypk9, and provide a comprehensive list of genes involved in manganese homeostasis. These data will empower further investigation into cellular mechanisms of manganese toxicity and the role of PARK9 in normal biology and neurodegenerative disease.

## Materials and Methods

### Yeast strains and culture

The *ypk9*Δ strain was obtained by replacing the YPK9 coding region with the *natMX* resistance cassette in the Y7092 (genotype: *can1delta::STE2pr-Sp_his5 lyp1delta his3delta1 leu2delta0 ura3delta0 met15delta0*) yeast strain background. Colony PCR was used to verify correct gene disruption. Cells were grown at 30°C on YPD or complete synthetic medium (CSM). Strains were manipulated and media prepared using standard techniques.

### Synthetic Genetic Array (SGA) screens

These screens were performed as described in [60], with some modifications, using a Singer RoToR HDA (Singer Instruments, Somerset, UK). To generate the query strain, the *natMX* cassette (confers resistance to clonNAT) was introduced into MATα strain Y7092 (gift from C. Boone). This query strain was mated to the yeast haploid deletion collection of non-essential genes (MATa, each gene deleted with KanMX cassette (confers resistance to G418)). The heterozygous diploids were transferred to sporulation media (low levels of carbon and nitrogen) and grown at room temperature for 5 days. The resulting spores were transferred for 2 days to synthetic medium lacking histidine (SD–His/Arg/Lys + canavanine, thialysine) to selectively germinate the *MAT*a meiotic progeny (this step was repeated twice). The *MAT*a meiotic progeny were transferred, by pinning, to agar plates containing kanamycin (SD/MSG–His/Arg/Lys + canavanine/thialysine/G418) to select for the MATa single-mutant progeny, and successively to agar plates containing both nourseouthricin and kanamycin (SD/MSG – His/Arg/Lys + canavanine/thialysine/G418/clonNAT) to select for growth of double-mutant haploids. The single and double selection plates (13 plates each containing 384 deletion mutants, spotted in duplicates) were photographed at day 3. The entire screen was repeated 3 times. The TS mutant library screen was performed in the same way, with a library of 1,335 temperature-sensitive mutants of essential genes [28], [29] spotted onto 5 plates with each strain spotted in quadruplicate. The plates were incubated at 25°C instead of 30°C. Colony sizes were measured using the ht-colony-measurer software [61]. The raw values were normalized dividing them by the median colony size of the plate. The data from 3 rounds were averaged and the difference between double and single mutant colony size was calculated. A threshold of −0.5 was used to call a synthetic sick/lethal interaction.

### Manganese tolerance screen

A total of 4799 mutant strains from the yeast haploid deletion collection of non-essential genes were pinned in duplicate on CSM plates containing G418 (200 mg/L) and 0, 12 and 25 mM MnCl_2_. The plates were grown at 30°C and photographed at day 3. The screen was repeated 3 times. Colony sizes were measured using the HT Colony Grid Analyzer software [61]. The raw values were normalized by the median colony size of the plate. Slow growing strains in 0 mM Mn^2+^ (with average colony size less than half of the plate median) and duplicate colonies that differed in size more than their average size were discarded from the analysis. Fitness was calculated dividing average colony size on Mn^2+^ containing plates by average colony size on control plates. We scored “sensitive” a strain with fitness <0.5 on 12 mM Mn^2+^ plates and “resistant” a strain with fitness >1.75 on 25 mM Mn^2+^, in 3 rounds of screen. To assess whether these phenotypes were dependent on Ypk9, we mated the library strains with the *ypk9*Δ strain (see SGA screen methods) and repeated the Mn^2+^ tolerance screen with the double mutant strains.

## Supporting Information

Table S1 (DOCX)

Table S2 (DOCX)
