# Supplementary material for: The Role of the Parkinson's Disease Gene PARK9 in Essential Cellular Pathways and the Manganese Homeostasis Network in Yeast
Source: PLoS One. 2012 Mar 23;7(3):e34178. doi: 10.1371/journal.pone.0034178 (PMC3311584; doi:10.1371/journal.pone.0034178)
Supplement: Table S1 — (DOCX) [file pone.0034178.s001.docx]

**Table S1.** Deletion strains sensitive to 12 mM Mn^2+^.

| **Phenotype** | **Yeast Gene** | **Human Homolog** | **Function** |
| --- | --- | --- | --- |
| Sensitive | APM1 | AP1M1 | Mu1-like medium subunit of the clathrin-associated protein complex |
| Sensitive | APQ12 |  | Protein required for nuclear envelope morphology, nuclear pore complex localization, mRNA export from the nucleus |
| Sensitive | APT1 | APRT | Adenine phosphoribosyltransferase |
| Sensitive | ARL1 | ARL1 | GTPase with a role in regulation of membrane traffic |
| Sensitive | ARP6 | ACTR6 | Actin-related protein that binds nucleosomes; a component of the SWR1 complex |
| Sensitive | ATG17 |  | Scaffold protein responsible for phagophore assembly site organization |
| Sensitive | ATP12 |  | Conserved protein required for assembly of alpha and beta subunits into the F1 sector of mitochondrial F1F0 ATP synthase |
| Sensitive | BRO1 | RHPN1 | Cytoplasmic class E vacuolar protein sorting (VPS) factor |
| Sensitive | BUD30 |  | Dubious open reading frame |
| Sensitive | CIK1 |  | Kinesin-associated protein required for both karyogamy and mitotic spindle organization |
| Sensitive | CLC1 |  | Clathrin light chain |
| Sensitive | CNB1 | TESC | Regulatory subunit of calcineurin |
| Sensitive | CRZ1 | ZNF705A | Transcription factor that activates transcription of genes involved in stress response |
| Sensitive | DIA2 |  | Origin-binding F-box protein that forms an SCF ubiquitin ligase complex |
| Sensitive | DOA1 | PLAA | WD repeat protein required for ubiquitin-mediated protein degradation |
| Sensitive | END3 |  | EH domain-containing protein involved in endocytosis, actin cytoskeletal organization and cell wall morphogenesis |
| Sensitive | GCS1 | ARFGAP1 | ARF GAP, involved in ER-Golgi transport; shares functional similarity with Glo3p |
| Sensitive | GLN3 | GATA1 | Transcriptional activator of genes regulated by nitrogen catabolite repression |
| Sensitive | GON7 |  | Component of the EKC/KEOPS protein complex |
| Sensitive | GOS1 | GOSR1 | v-SNARE protein involved in Golgi transport |
| Sensitive | HIT1 |  | Protein of unknown function |
| Sensitive | HTL1 |  | Component of the RSC chromatin remodeling complex |
| Sensitive | HTZ1 | H2AFZ | Histone variant H2AZ, exchanged for histone H2A in nucleosomes by the SWR1 complex |
| Sensitive | IRS4 |  | EH domain-containing protein involved in regulating phosphatidylinositol 4,5-bisphosphate levels and autophagy |
| Sensitive | LPP1 |  | Lipid phosphate phosphatase |
| Sensitive | LYS5 |  | Phosphopantetheinyl transferase involved in lysine biosynthesis |
| Sensitive | MNR2 |  | Vacuolar membrane protein required for magnesium homeostasis |
| Sensitive | MOG1 | RANGRF | Conserved nuclear protein that stimulates nucleotide release, involved in nuclear protein import |
| Sensitive | NNF2 |  | Protein that exhibits physical and genetic interactions with Rpb8p, which is a subunit of RNA polymerases I, II, and II |
| Sensitive | NPL3 |  | RNA-binding protein required for pre-mRNA splicing |
| Sensitive | PKR1 |  | V-ATPase assembly factor |
| Sensitive | POP2 | CNOT7 | RNase of the DEDD superfamily |
| Sensitive | PPQ1 |  | Putative protein serine/threonine phosphatase |
| Sensitive | PUN1 |  | Putative protein of unknown function |
| Sensitive | RAV1 | DMXL1 | Subunit of the RAVE complex, which promotes assembly of the V-ATPase holoenzyme |
| Sensitive | RGP1 |  | Subunit of a Golgi membrane exchange factor (Ric1p-Rgp1p) that catalyzes nucleotide exchange on Ypt6p |
| Sensitive | RIC1 |  | Protein involved in retrograde transport to the cis-Golgi network; forms heterodimer with Rgp1p |
| Sensitive | RPB9 | POLR2I | RNA polymerase II subunit |
| Sensitive | RPL31A | RPL31 | Protein component of the large (60S) ribosomal subunit |
| Sensitive | RPP1A | RPLP1 | Ribosomal stalk protein P1 alpha |
| Sensitive | RVS161 | BIN3 | Amphiphysin-like lipid raft protein |
| Sensitive | SEC22 | SEC22A | R-SNARE protein |
| Sensitive | SNF6 |  | Subunit of the SWI/SNF chromatin remodeling complex |
| Sensitive | SWA2 |  | Auxilin-like protein involved in vesicular transport |
| Sensitive | SWC3 |  | Component of the SWR1 complex, which exchanges histone variant H2AZ (Htz1p) for chromatin-bound histone H2A |
| Sensitive | SWC5 | CFDP1 | Component of the SWR1 complex, which exchanges histone variant H2AZ (Htz1p) for chromatin-bound histone H2A |
| Sensitive | SWI3 | SMARCC1 | Subunit of the SWI/SNF chromatin remodeling complex |
| Sensitive | SWR1 | SRCAP | Swi2/Snf2-related ATPase that is the structural component of the SWR1 complex, which exchanges histone variant H2AZ (Htz1p) for chromatin-bound histone H2A |
| Sensitive | SYS1 |  | Integral membrane protein of the Golgi required for targeting of the Arf-like GTPase Arl3p to the Golgi |
| Sensitive | TEF4 | EEF1G | Gamma subunit of translational elongation factor eEF1B |
| Sensitive | TLG2 | STX16 | Syntaxin-like t-SNARE |
| Sensitive | UME6 |  | Key transcriptional regulator of early meiotic genes |
| Sensitive | VMA21 |  | Integral membrane protein that is required for vacuolar H+-ATPase (V-ATPase) function |
| Sensitive | VPS1 | DNM1 | Dynamin-like GTPase required for vacuolar sorting |
| Sensitive | VPS51 |  | Component of the GARP (Golgi-associated retrograde protein) complex |
| Sensitive | VPS52 | VPS52 | Component of the GARP (Golgi-associated retrograde protein) complex |
| Sensitive | VPS53 | VPS53 | Component of the GARP (Golgi-associated retrograde protein) complex |
| Sensitive | VPS54 |  | Component of the GARP (Golgi-associated retrograde protein) complex |
| Sensitive | VPS61 |  | Dubious open reading frame |
| Sensitive | VPS63 |  | Dubious open reading frame, 98% of ORF overlaps the verified gene YPT6 |
| Sensitive | VPS64 |  | Protein required for cytoplasm to vacuole targeting of proteins |
| Sensitive | VPS71 |  | Nucleosome-binding component of the SWR1 complex |
| Sensitive | VPS72 |  | Htz1p-binding component of the SWR1 complex |
| Sensitive | YAF9 | YEATS2 | Subunit of both the NuA4 histone H4 acetyltransferase complex and the SWR1 complex |
| Sensitive | YLR402W |  | Dubious open reading frame |
| Sensitive | YMR031W-A |  | Dubious open reading frame unlikely to encode a protein, partially overlaps the uncharacterized ORF YMR031C |
| Sensitive | YPT6 | RAB6A | Rab family GTPase, Ras-like GTP binding protein involved in the secretory pathway |
