# Supplementary material for: The Role of the Parkinson's Disease Gene PARK9 in Essential Cellular Pathways and the Manganese Homeostasis Network in Yeast
Source: PLoS One. 2012 Mar 23;7(3):e34178. doi: 10.1371/journal.pone.0034178 (PMC3311584; doi:10.1371/journal.pone.0034178)
Supplement: Table S2 — (DOCX) [file pone.0034178.s002.docx]

**Table S2.** Deletion strains resistant to 25 mM Mn^2+^.

| **Phenotype** | **Yeast Gene** | **Human Homolog** | **Function** |
| --- | --- | --- | --- |
| Resistant | ADE5,7 | GART | Enzyme of the 'de novo' purine nucleotide biosynthetic pathway |
| Resistant | ATP5 | ATP5O | Subunit 5 of the stator stalk of mitochondrial F1F0 ATP synthase |
| Resistant | BLM10 | PSME4 | Proteasome activator subunit |
| Resistant | COA1 |  | Mitochondrial inner membrane protein required for assembly of the cytochrome c oxidase complex |
| Resistant | CPR7 |  | Peptidyl-prolyl cis-trans isomerase (cyclophilin) |
| Resistant | CSF1 | KIAA1109 | Protein required for fermentation at low temperature |
| Resistant | EGD2 | NACA | α subunit of the heteromeric nascent polypeptide-associated complex (NAC) |
| Resistant | ERD1 |  | Predicted membrane protein required for the retention of lumenal endoplasmic reticulum proteins |
| Resistant | ERG3 | SC5DL | C-5 sterol desaturase |
| Resistant | FRA1 | XPNPEP1 | Protein involved in negative regulation of transcription of iron regulon |
| Resistant | FRA2 | BOLA2 | Protein involved in negative regulation of transcription of iron regulon |
| Resistant | GGA2 | GGA2 | Protein that interacts with and regulates Arf1p and Arf2p in a GTP-dependent manner to facilitate traffic through the late Golgi |
| Resistant | GRX7 |  | Cis-golgi localized monothiol glutaredoxin |
| Resistant | HDA1 | HDAC9 | Putative catalytic subunit of a class II histone deacetylase complex that also contains Hda2p and Hda3p |
| Resistant | HDA2 |  | Subunit of a class II histone deacetylase complex |
| Resistant | HDA3 |  | Subunit of a class II histone deacetylase complex |
| Resistant | IMP2' |  | Transcriptional activator involved in maintenance of ion homeostasis |
| Resistant | KRE28 |  | Subunit of a kinetochore-microtubule binding complex |
| Resistant | LDB18 |  | Component of the dynactin complex, which is required for dynein activity |
| Resistant | MAM3 | CNNM1 | Protein required for normal mitochondrial morphology |
| Resistant | MDM32 |  | Mitochondrial inner membrane protein, required for normal mitochondrial morphology and inheritance |
| Resistant | MGM1 | OPA1 | Mitochondrial GTPase, required for mt morphology and genome maintenance |
| Resistant | MGM101 |  | Protein involved in mitochondrial genome maintenance |
| Resistant | MNN9 |  | Subunit of Golgi mannosyltransferase complex |
| Resistant | MRPL10 | MRPL15 | Mitochondrial ribosomal protein of the large subunit |
| Resistant | MRPL37 |  | Mitochondrial ribosomal protein of the large subunit |
| Resistant | MRPL6 |  | Mitochondrial ribosomal protein of the large subunit |
| Resistant | MSL1 | SNRPA | U2B component of U2 snRNP, involved in splicing |
| Resistant | NEM1 | CTDNEP1 | Probable catalytic subunit of Nem1p-Spo7p phosphatase holoenzyme |
| Resistant | NRP1 |  | Putative RNA binding protein of unknown function |
| Resistant | NYV1 |  | v-SNARE component of the vacuolar SNARE complex involved in vesicle fusion |
| Resistant | PAN6 |  | Pantothenate synthase |
| Resistant | PHO87 | SLC13A1 | Low-affinity inorganic phosphate transporter |
| Resistant | PMT1 |  | Protein O-mannosyltransferase |
| Resistant | PPM1 |  | Carboxyl methyltransferase |
| Resistant | QDR2 |  | Multidrug transporter of the major facilitator superfamily |
| Resistant | RPS12 | RPS12 | Protein component of the small (40S) ribosomal subunit |
| Resistant | RRG1 |  | Protein of unknown function, required for vacuolar acidification and mitochondrial genome maintenance |
| Resistant | RRT7 |  | Dubious open reading frame |
| Resistant | RSM23 |  | Mitochondrial ribosomal protein of the small subunit |
| Resistant | RSM7 |  | Mitochondrial ribosomal protein of the small subunit |
| Resistant | RTG2 |  | Sensor of mitochondrial dysfunction |
| Resistant | RTT103 | RPRD1A | Protein that plays a role in transcription termination by RNA polymerase II |
| Resistant | SAP190 | PPP6R1 | Protein that forms a complex with the Sit4p protein phosphatase and is required for its function |
| Resistant | SGO1 |  | Component of the spindle checkpoint |
| Resistant | SHR5 |  | Subunit of a palmitoyltransferase |
| Resistant | SLM5 | NARS2 | Mitochondrial asparaginyl-tRNA synthetase |
| Resistant | SLS1 |  | Mitochondrial membrane protein that coordinates expression of mitochondrially-encoded genes |
| Resistant | SRV2 | CAP1 | CAP (cyclase-associated protein) subunit of adenylyl cyclase complex |
| Resistant | SSE1 | HSPA4 | ATPase that is a component of the heat shock protein Hsp90 chaperone complex |
| Resistant | SUR2 |  | Sphinganine C4-hydroxylase |
| Resistant | SUR4 | ELOVL1 | Elongase, involved in fatty acid and sphingolipid biosynthesis |
| Resistant | SYG1 | XPR1 | Plasma membrane protein of unknown function |
| Resistant | TIP41 | TIPRL | Component of the TOR signaling pathway |
| Resistant | TUF1 | TUFM | Mitochondrial translation elongation factor Tu |
| Resistant | UBP1 | USP30 | Ubiquitin-specific protease |
| Resistant | VPS9 | GAPVD1 | A guanine nucleotide exchange factor involved in vesicle-mediated vacuolar protein transport |
| Resistant | VTC1 |  | Subunit of the vacuolar transporter chaperone (VTC) complex |
| Resistant | VTC3 |  | Subunit of the vacuolar transporter chaperone (VTC) complex |
| Resistant | VTC4 |  | Vacuolar membrane polyphosphate polymerase; subunit of the vacuolar transporter chaperone (VTC) complex |
| Resistant | YCK1 | VRK1 | Palmitoylated plasma membrane-bound casein kinase I isoform |
| Resistant | YDR089W |  | Unknown function; deletion confers resistance to Nickel |
| Resistant | YEL057C |  | Protein of unknown function involved in telomere maintenance |
| Resistant | YGR102C |  | Subunit of the trimeric GatFAB AmidoTransferase(AdT) complex |
| Resistant | YHR180W |  | Dubious open reading frame |
| Resistant | YIL054W |  | Dubious open reading frame |
| Resistant | YLL044W |  | Dubious open reading frame |
| Resistant | YLR001C |  | Putative protein of unknown function |
| Resistant | YML020W |  | Putative protein of unknown function |
| Resistant | YOR199W |  | Dubious open reading frame |
| Resistant | YOR200W |  | Dubious open reading frame |
| Resistant | ZEO1 |  | Peripheral membrane protein of the plasma membrane |
